# Lithium placental passage at delivery: an observational study

**DOI:** 10.1192/j.eurpsy.2022.1017

**Published:** 2022-09-01

**Authors:** M.L. Imaz, L. Garcia-Esteve, M. Torra, D. Soy, K. Langohr, R. Martin-Santos

**Affiliations:** 1 Hospital Clinic, Unit Of Perinatal Mental Health Clínic-bcn. Department Of Psychiatry And Psychology, Barcelona, Spain; 2 Hospital Clinic, Pharmacology And Toxicology Laboratory, Biochemistry And Molecular Genetics Service, Biomedical Diagnostic Center (cbd), Barcelona, Spain; 3 Hospital Clinic, Division Of Medicines. Department Of Pharmacy, Barcelona, Spain; 4 Universitat Politècnica de Catalunya, Statistics And Operations Research, Barcelona, Spain; 5 Hospital Clínic, Department Of Psychiatry And Psychology, Barcelona, Spain

**Keywords:** Placental passage, Mother-infant pair, Lithium blood levels, Delivery

## Abstract

**Introduction:**

Lithium is used as a first-line treatment for bipolar disorder during perinatal period. Dosing of lithium can be challenging as a result of pharmacokinetic changes in renal physiology. Frequent monitoring of lithium blood levels during pregnancy is recommended in order remain within the therapeutic window (0.5 to 1.2 mEq/L). Lower neonatal lithium blood level (<0.64 mEq/L) at time of delivery reduces the risk of lithium side effects in the neonate.

**Objectives:**

The aim of the present study was to quantify the rate of lithium placental passage in real word.

**Methods:**

We included a total of 68 mother-infant pairs for which a lithium measurement was performed intrapartum. Lithium serum concentrations were determined by means of an AVL 9180 electrolyte analyzer. The limit of quantification (LoQ) was 0.20 mEq/L and detection limit was 0.10 mEq/L. Pearson analyse was performer to assess the correlation between mother and umbilical cord lithium serum concentrations.

**Results:**

The mean of umbilical cord serum concentration at delivery was 0.57 mEq/L (SD=0.26, range 0,20-1,42). The mean infant-mother lithium ratio at delivery for the 68 pairs was 1.12 (SD=0.24) across a wide range of maternal concentrations (range 0.14-1,40 mEq/L). There was a strong positive correlation between maternal and umbilical cord lithium blood levels (Peearson correlation coefficient 0.948, p<0.001).

**Conclusions:**

Lithium demostrates complete placental passage. This finding is consistent with the results of others studies (Newport 2005; Molenaar 2021).

**Disclosure:**

No significant relationships.

